# Bringing computational science to the public

**DOI:** 10.1186/s40064-016-1856-7

**Published:** 2016-03-02

**Authors:** James L. McDonagh, Daniel Barker, Rosanna G. Alderson

**Affiliations:** Biomedical Sciences Research Complex and EaStCHEM School of Chemistry, University of St Andrews, Purdie Building, North Haugh, St Andrews, KY16 9ST Fife UK; Manchester Institute of Biotechnology, The University of Manchester, 131 Princess Street, Manchester, M1 7DN UK; Sir Harold Mitchell Building, School of Biology, University of St Andrews, St Andrews, Fife, KY16 9TH UK; Institute of Evolutionary Biology, University of Edinburgh, Charlotte Auerbach Road, The Kings Buildings, Edinburgh, EH9 3FL UK

**Keywords:** Computational science, Education, Bioinformatics, 4273*π*, 4273pi, Public events, Teaching, Public engagement

## Abstract

**Background:**

The increasing use of computers in science allows for the scientific analyses of large datasets at an increasing pace. We provided examples and interactive demonstrations at Dundee Science Centre as part of the 2015 Women in Science festival, to present aspects of computational science to the general public. We used low-cost Raspberry Pi computers to provide hands on experience in computer programming and demonstrated the application of computers to biology. Computer games were used as a means to introduce computers to younger visitors. The success of the event was evaluated by voluntary feedback forms completed by visitors, in conjunction with our own self-evaluation. This work builds on the original work of the 4273*π* bioinformatics education program of Barker et al. (2013, BMC Bioinform. 14:243). 4273*π* provides open source education materials in bioinformatics. This work looks at the potential to adapt similar materials for public engagement events.

**Results:**

It appears, at least in our small sample of visitors (n = 13), that basic computational science can be conveyed to people of all ages by means of interactive demonstrations. Children as young as five were able to successfully edit simple computer programs with supervision. This was, in many cases, their first experience of computer programming. The feedback is predominantly positive, showing strong support for improving computational science education, but also included suggestions for improvement.

**Conclusions:**

Our conclusions are necessarily preliminary. However, feedback forms suggest methods were generally well received among the participants; “*Easy to follow. Clear explanation*” and “*Very easy. Demonstrators were very informative.*” Our event, held at a local Science Centre in Dundee, demonstrates that computer games and programming activities suitable for young children can be performed alongside a more specialised and applied introduction to computational science for older visitors.

**Electronic supplementary material:**

The online version of this article (doi:10.1186/s40064-016-1856-7) contains supplementary material, which is available to authorized users.

## Background

Computation is widely used in science, technology, engineering, mathematics and medicine (STEMM) subjects. The use of command line computation to run standard computational analyses is becoming, increasingly, as important as other field-specific skills. Recent efforts have improved computational science at schools in the UK (Gallagher et al. 2011; Leach et al. 2006; Wood and Gebhardt 2013; Barker et al. 2015), although there remains room for improvement (Furber 2012; Weale 2015). We classify computational science here as distinct from computer science. Here, we define computational science as being the application and use of computational knowledge and skills (including programming) in order to aid in the understanding of and make new discoveries in traditional sciences, as opposed to the study of computers and computing *per se*. Most computational science involves the use of UNIX or Linux operating systems and command-line computing, as commonly used on high performance computing (HPC) clusters. This is in contrast to the graphical user interfaces (GUIs) and apps pupils are often more familiar with—for example, from school-level ICT under old curricula in the UK (Furber 2012), mobile phones and home entertainment systems. Despite it’s ‘alien’ interface, it seems command line computing may be rapidly picked up by young people without much training (Barker et al. 2015). This is encouraging for developing computational skills for future scientists. In recent years, we have seen recognition of the importance of computer skills in the UK education system. In England and Wales computer programming is introduced in primary schools (Department for Education UK 2013). In Scotland there has been a drive to introduce it at early secondary school level (Royal Society of Edinburgh) and it also appears later, *via* bioinformatics in Higher Biology (Scottish qualifications authority 2014).

Despite these educational initiatives, we see a requirement for public engagement in computational science. Firstly, this will assist young visitors, potentially enthuse them, and provide context for computational education received at school. Secondly, older visitors may have been educated under older curricula, in which computational science is completely absent. A drop-in event allows interested members of both audiences to be reached. We created and delivered an event that introduced computational science by means of an interactive workshop at a regional science centre. We aimed to increase public awareness of computational research in STEMM subjects. Although some computational science requires large, expensive computer clusters, other aspects are amenable to low-cost home computers or cheap cloud services, accessible to amateurs once sufficient background knowledge has been attained. In this way, we hope to contribute to the longer-term possibility of democratisation of science. The ‘passing trade’ style of the event and the young age of many of the children meant the emphasis tended to more basic computer programming and games, although opportunities arose to elaborate on more specific applications of computational science especially with the older visitors. The computational science materials were based on 4273*π* (Barker et al. 2013; 4273*π*^2015^).

## Event details

**Fig. 1:**
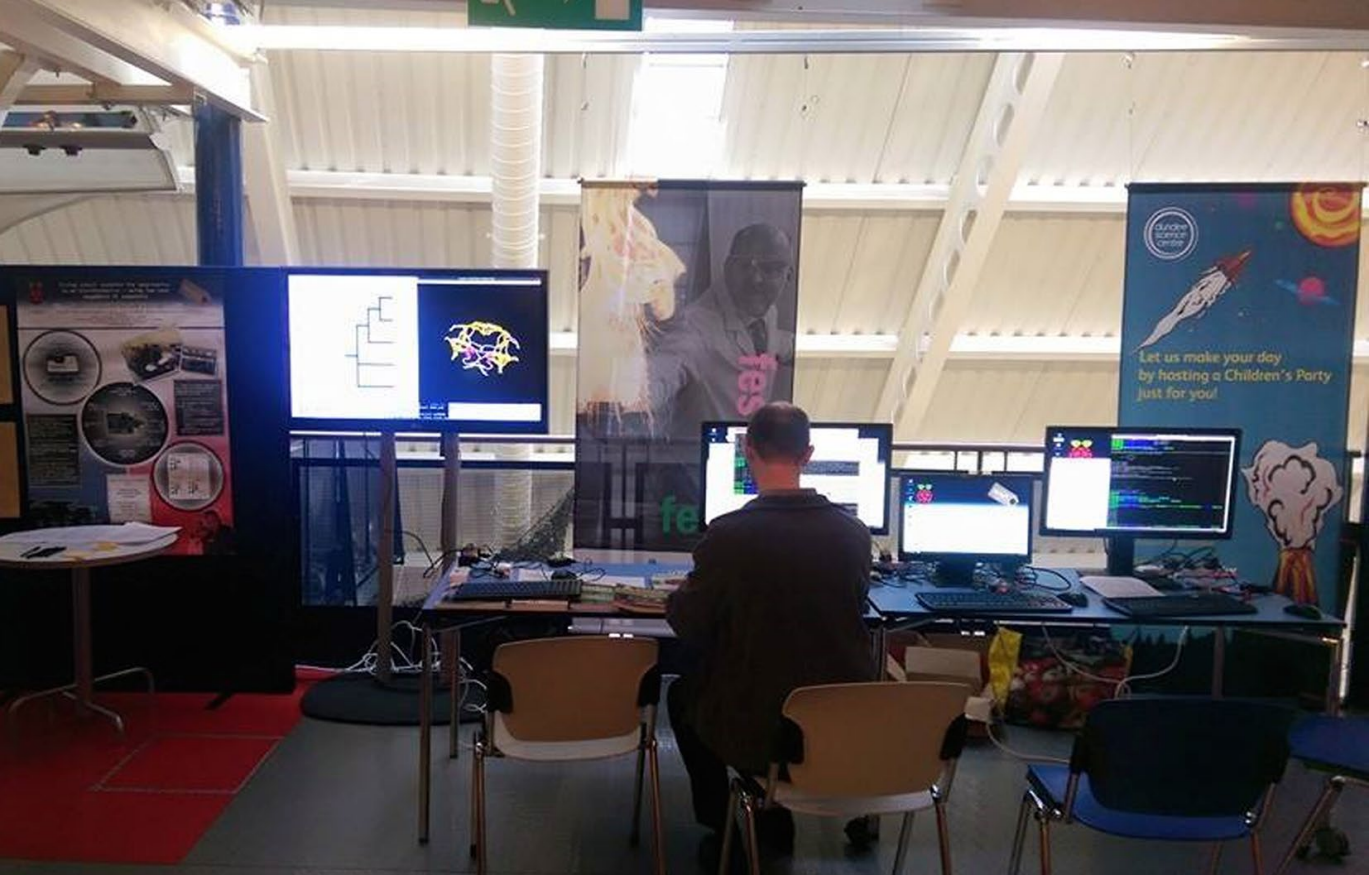
Interactive activity space used in the Dundee Science Centre

In this work, we report the findings from an interactive programming event held at the Dundee Science Centre as part of the Women in Science Festival 2015 (Fig. 1). On Pi day (14th March) 2015, the authors took four Raspberry Pi computers (Raspberry Pi Foundation), examples of the teaching material, reference guides and a previously presented poster (Alderson 2014) detailing the main aims and previous results of the project. We divided our exhibition into different ‘stations’, in which visitors were given a choice from a range of guided activities designed by ourselves. These included basic scripting in the Bash, Python or Perl languages and interactive games. We left it up to each individual visitor to decide which activity they wanted to try. Whilst carrying out their activity, visitors were shown and handled the Raspberry Pi computer. It was explained to them the range of activities that was possible using the device. Visitors were then shown the poster and monitor display showing the applications of in silico experiments in biology. There was a wide variety of participants, with a range of ages and social backgrounds being attracted to the event. Young children played several python-based games (squirrel.py and snake.py) before being shown the source code as a means to practically introduce how computer applications worked (Long 2007). This enabled even very young children to gain the non-intuitive concept that lines of commands can produce rules and graphics enabling the development of computer applications. Adults, parents and interested children were introduced via demonstrations to some of the concepts of computational biology including biologically interesting examples of protein structure, phylogeny (demonstration based on FOXP2 proteins) and genome annotation. As a preliminary study, we present the analysis of the 13 feedback forms, voluntarily filled in by visitors (Additional files 1, 2, 3).

## Results and discussion

Below we present tables of the raw responses of the 13 completed feedback forms, along with summary graphs for the relevant responses. The full feedback form is provided in Additional file 1. Raw responses are in Additional file 3 and statistical calculations are in Additional file 2. The feedback form consists of 13 questions in total; three general (yes, no type) questions, concerning a person’s background, eight Likert scale questions to rate the workshop and two free text questions to make suggestions or further comment on the workshop. Our choice of questions was deliberately basic, the questionnaires and analysis being a preliminary step to set the foundation for future studies. We plan to use the results as a baseline on which to design future workshops and methods of quantitative and qualitative evaluation.

**Fig. 2:**
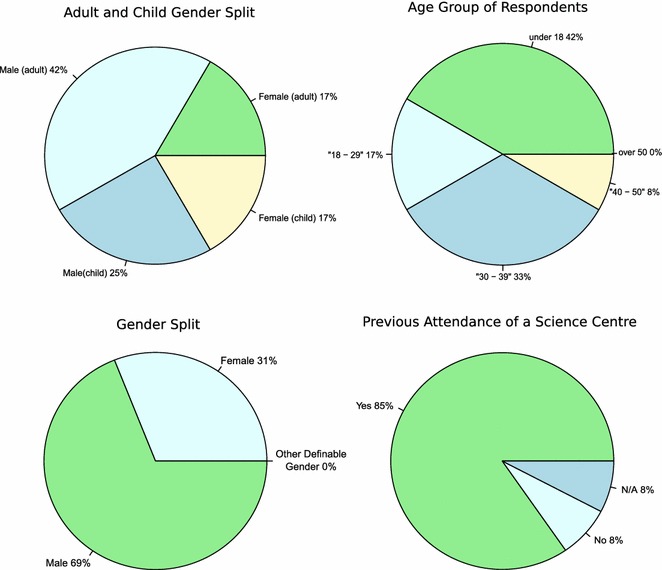
Pie chart plots summarising the data collected about the background of the visitors. Proportions for the pie charts ‘Adult child gender split’ and ‘Gender split’ differ, because one participant chose not to give their age

We initially take the questions on background for analysis of who was attending the workshop. This section consists of three questions: “*Have you attended a science centre before”* with a yes or no response. The second question “*What is your gender?*” With possible responses being female, male or a self-defined ‘other’ category (Balarajan et al. 2011). The final question in this section is “*What is your age?*”, with an open response for the persons age. Children were defined as under 18, and adults as 18 and over. Table 1; Fig. 2 summarise the results.

**Table 1:** The Likert questions and a count of the responses given by the visitors

Question#	Question	Strongly disagree	Disagree	Neutral	Agree	Strongly agree
Q1	I am interested in science	0	0	1	4	8
Q2	Science experiments can be carried out on computers	0	0	1	3	9
Q3	I use a computer more than a tablet or other hand held device	1	3	1	1	7
Q4	Having attended the workshop today I am less excited by science	5	3	2	2	1
Q5	Having attended the workshop today I think computers are useful within biology	0	0	1	3	9
Q6	Having attended the workshop today I am more interested in programming a computer	0	0	1	5	7
Q7	Having attended the workshop today I am likely to use a Raspberry Pi in my own time	0	2	5	2	4
Q8	Having attended the workshop today I believe this type of workshop would be useful for students aged between 15 and 18	0	0	2	5	6

The gender breakdown suggests approximately a two-third majority were male, compared to a third female (Fig. 1; Table 1; under a binomial distribution, for observed proportion female vs an extrinsic hypothesis of 0.5 female, likelihood ratio 0.37, constituting weak evidence for a gender imbalance). We can also see an age range of 40 years, with the youngest being five and the eldest forty-five. It is also noteworthy that the vast majority had attended a science centre before this event, so the majority of visitors are likely already interested in science as a whole. It is fair to assert on the basis of these results we were reaching a diverse audience in terms of gender and age, but an audience likely already interested in science. The gender imbalance is numerically more pronounced in the over 18 age groups, although the evidence is again weak (likelihood ratio 0.51). In contrast, the gender gap is numerically much narrower for the under 18 group, with correspondingly weaker evidence for a gender imbalance (likelihood ratio 0.90). Given the small sample size, leading to low statistical power, these results must be considered approximate. Care must also be taken in interpreting the age answers in the feedback forms as it is unclear whether an adult has filled in the form on the child’s behalf, therefore, we cannot be sure as to what extent the answers are actually the adult’s perception of the child’s experience.

**Fig. 3:**
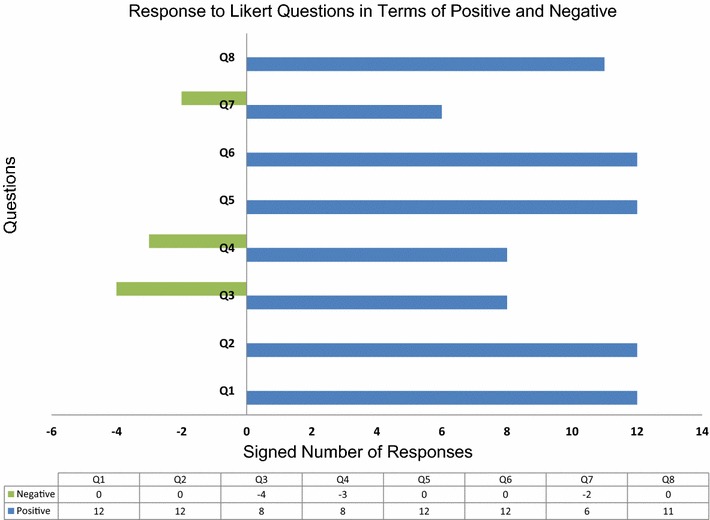
Responses to the Likert questions are catagorised here  as agreeing or disagreeing with the question. Neutral answers were removed, so count as neither positive nor negative

**Fig. 4:**
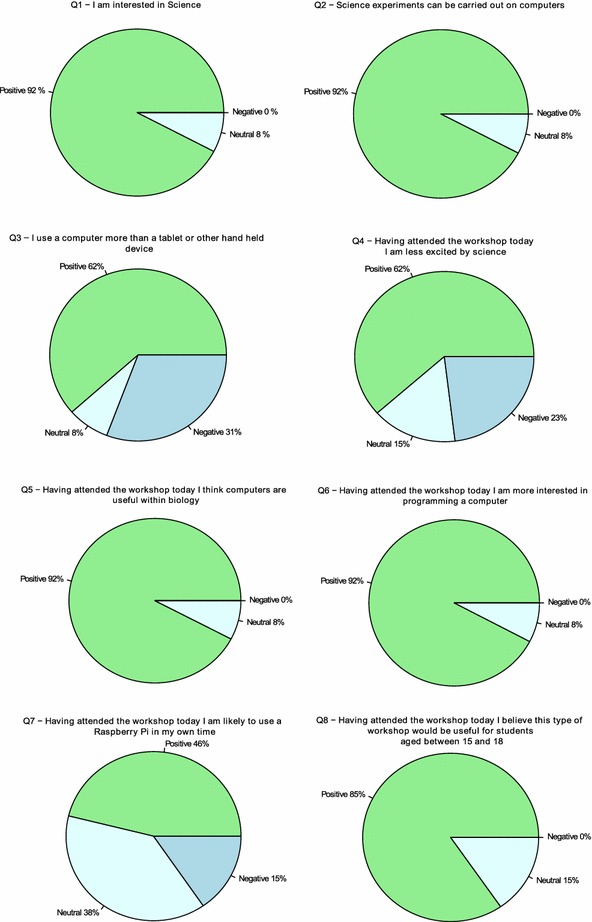
Responses to the Likert questions are summarised as *pie charts* showing the proportions of responses in each category

In the following, Table 1 gives the questions and a count of the responses from visitors. This is summarised in Figs. 3, 4.

The Likert questions were constructed so that the sense of the question was not consistent throughout the questionnaire. This was to assist in the detection of someone simply ticking one answer category consistently for all questions. These eight questions were selected to provide us with specific information about the workshop. The first two questions provide further background data about the visitors: Q1 queries if the visitor has a general interest in science, where we test our assumption that this would be the case for most science centre visitors. Q2 assess if the visitors are generally aware of in silico science experiments. Q3 informs us of a person’s computational background. Q4 begins the section of questions designed to feedback on how well the workshop was received. Q4 tests whether we have reduced a person’s enthusiasm for science. In Q5 we test how well we have communicated the usefulness of computation in biology. Q6 is designed to check how well received information  regarding computer programming had been. Q7 tests whether the workshop has encouraged the public to consider self-learning. Finally, Q8, is used to gauge the public response to our goal of introducing such workshops into schools. This is a useful question to assess the degree of public support for a programme such as 4273*π*. Figs 3, 4 summarise the results given in Table 1.

In Figs. 3, 4, we can see clearly that in all cases the members of the public who filled in the feedback forms agreed with what we would consider to be the positive sense of the Likert questions. We consider either strongly agreeing or agreeing with Q1 (“I am interested in Science”), Q2 (“Science experiments can be carried out on computers”), Q5 (“Having attended the workshop today I think computers are useful within biology”), Q6 (“Having attended the workshop today I am more interested in programming a computer”), Q7 (“Having attended the workshop today I am likely to use a Raspberry Pi in my own time”) or Q8 (“Having attended the workshop today I believe this type of workshop would be useful for students aged between 15 and 18″) and either strongly disagreeing or disagreeing with Q3 (“I use a computer more than a tablet or other hand held device”) or Q4 (“Having attended the workshop today I am less excited by science”) to be a positive response. From our results, it is clear that Q3, Q4 and Q7 were controversial in their answers.

Q3 (“I use a computer more than a tablet or other hand held device”) refers to the visitor’s background and their preference for a computer over a handheld or portable device. The result suggests most people still use computers over hand held or other portable devices, such as tablets. An interesting point is that although we see a mix of ages agreeing or strongly agreeing with this question, no single age group is dominant. However, for those who either disagree or strongly disagree, there is a dominant age group. Half of the negative responses come from the under 18 age group.

Q7 (“Having attended the workshop today I am likely to use a Raspberry Pi in my own time”) refers to how many people the workshop has inspired to consider self-study of bioinformatics and computational science, using a Raspberry Pi. We would not have expected everyone to be willing to undertake such a task after only one workshop. The fact that the responses suggest more people have been encouraged to consider self-study than have remained with the *status quo* is a very positive result. In fact, several members of the public enquired where to purchase a Raspberry Pi and where to find reference guides.

From our point of view, Q4 (“Having attended the workshop today I am less excited by science”) is a disappointing result. Our perception from the public was positive, with many visitors actively engaged in the tasks. On the face of it, this result suggests that several people left the workshop less excited by science. We were provided with some verbal feedback suggesting that the children in particular struggled with the shift of the positive and negative sense in the Likert questions. Hence, the negative response could be largely or entirely due to a misreading of the sense of the question by respondents. Two of the three respondents who stated that they either strongly agreed or agreed with Q4 were children. Additionally, we note that despite the respondent’s negative response here, all respondents gave a positive response to at least one of Q6 (“Having attended the workshop today I am more interested in programming a computer”) and Q8 (“Having attended the workshop today I believe this type of workshop would be useful for students aged between 15 and 18”). One may expect to see negative responses to Q6 and Q8 if a respondent gave a negative response to Q4. One of these respondents also left a free text comment simply stating “No” to the free text question: “Do you have any suggestions to improve the workshop?” implying they were either satisfied with the workshop, not fully satisfied but without suggestion for improvement or saw no future for it. Whilst we cannot, at this stage, determine the reasons for this result, the evidence, when we consider responses to other questions, reveals a possibility that the question has been misread/misinterpreted. Finally, we suggest that—if some respondents decided against pursuit of science as a result of greater experience of science—this is, on a broader level, a positive outcome. Non-STEMM subjects are important and an informed decision to focus on these, rather than STEMM, would be a successful outcome. However, we are unable to distinguish among the possible reasons for the outcome of Q4.

More generally, we also note the difficulty of presenting to such a mixed audience; especially when the materials and concepts are alien to most of their everyday lives (Teske 2015). Although presentation of material was specifically designed to be adapted to a wide range of visitor ages, it is not possible to design material equally acceptable across all ages (Teske 2015). This was suggested in some of the free text feedback, “*Very easy for me*. *Primary age children need more time I think*”. For future events, we will look to include more age defined tasks and include activities in which the complexity level is more amenable to user adjustment. A further suggestion is that the workshop could be more visually, kinaesthetically and audibly engaging, for example, as suggested in one free text response “*More whizzbang type stuff*”.

The free text questions left space for visitors to leave overall comments on the workshop and propose improvements. We were given two proposals: The first, as discussed above, “*More whizzbang type stuff*” and the second, “*would like to spend more time learning about it and hope to read some of the raspberry pi books/articles with better understanding now*.” As we stated above we will look at including some improved visual, kinaesthetic and auditory aids in future workshops and perhaps employ a hands-on workshop and tutorial model. This may then leave more time to spend with small groups who are eager to learn more. In terms of the comments on the overall workshop, the reactions were generally positive, for example: “*Easy to follow. Clear explanation*” and “*Very easy. Demonstrators were very informative.*” There were several mixed answers, for example: “*It was okay but a litt[l]e bit confusing of parts*” and “*Very easy*”. For the latter comment, it is unclear whether this is to be taken as positive or negative, but taking the two mixed statements together lends support for our earlier statements about the difficulties of presenting such material in an accessible and suitable way to a broad audience.

This analysis serves as a useful starting point for future study. In future work we intend to make better use of the rich information available in the free text responses by designing quantitative measures of positive and negative word counting for an enhanced analysis (Ryan and Bernard 2003). We can similarly produce a list of words we may wish to see in the responses, specifically related to the workshop outcomes, and count the occurrences of these. Additionally, the analysis could be augmented with qualitative analysis of the free text similar to that presented here. It would be useful to implement short, on the spot, interviews. These could be combined enhanced with optional, long-term follow-up with older participants by email, to evaluate whether the activity led to any long-term changes in behaviour.

Our own perceptions of the event were positive. The experience was very engaging and enlightening. Our audience’s curiosity permitted us to describe our academic research area and in doing so explain why computational science is an important research area. This lead naturally to conversations about the importance of teaching computer skills in schools for the next generation. Our reply to the audience’s curiosity came through varied means, including conversation, interactive activities and examples. It seems our message was well received, judging by the feedback above. The public were open and enthusiastic, providing us with plenty of useful feedback which we will take note of and seek to address in future workshops.

Since this event we have planned and hosted another workshop in which we responded to some of the comments made here. The workshop was run simultaneously at two sites, at the Medical and Biological Sciences Building at the University of St Andrews and the Manchester Museum at the University of Manchester, as part of larger public events on European Researchers’ night (at St Andrews, part of the ResearchLive! Programme and at Manchester part of Science Uncovered). We employed a bioinformatics-inspired citizen science game, Phylo (Kawrykow et al. 2012), which enabled a continuous discussion of the topic whilst practically engaging the participants. We set up a competition between the two sites, based on the best cumulative score on Phylo. At Manchester we additionally employed the SCRATCH introductory programming language to introduce concepts more easily to younger participants. These changes provided improved visual and audio engagement through the tasks presented and the competition.

We are currently considering options to organise further workshops, and will combine the data from a number of workshops to provide a larger sample and, we hope, more generalizable conclusions. We hope to include on the spot interviews and longer-term contact in future workshops.

## Conclusions

Within the limited scope of this study (small sample size, single event, assessed by one-off questionnaires only), we see strong positive results from most aspects of the workshop. Our main aim in carrying out this activity was to increase awareness of computational research in STEMM subjects, principally biology. Q5 (“Having attended the workshop today I think computers are useful within biology”) of the Likert questions was designed to test this. 69 % (9 people) of respondents strongly agreed with this statement a further 23 % (3 people) agreed and 8 % (1 person) neutral. This information suggests our primary aim was achieved.

We were additionally interested in the level of public support for teaching these skills to young adults through similar workshops, the premise for 4273*π*. There appears strong support from the public for such workshops to be available to students aged 15–18 (Q8)—a major aim of the 4273*π* project. We assign this question as having strong support, due to the receipt of no negative answers.

Q3 poses a potentially interesting conclusion. The data shows that among those who disagree or strongly disagree with the statement, “I use a computer more than tablet or other hand held device”, half come from the under 18 group. For those in support of the statement, the age range is more widely distributed. Although the numbers are too small to draw firm conclusions, this suggests we might have to do more to encourage computer use over portable or hand held devices in the future. Alternatively, should computational science move to hand-held devices, it would be possible to deliver computational science to the public on such hardware.

Most visitors seemed to enjoy learning to program and the application of bioinformatics to medically relevant problems (Q5 and Q6). We cannot rule out that the negative results stem from format issues, firstly, the use of feedback forms in which we alternate positive and negative sense questions may have caused confusion, especially for younger visitors. From conversations with visitors, our own perception is that two questionnaires aimed at the different age groups may have been more beneficial, as some of the younger visitors struggled with the swapping of sense on the questions. Secondly, the event format requires some revision. A greater flexibility in the tasks is required, allowing visitors to advance the complexity of the task at their own pace. As we suggest above, a hands-on workshop, similar to that which is provided here, but coupled with short tutorial demonstrations, may provide a better structure. This is something we will look to address for future events.

## Additional files

10.1186/s40064-016-1856-7 Raspberry pi and bioinformatics in the Dundee science centre questionnaire.
10.1186/s40064-016-1856-7 Calculation of likelihood ratios.
10.1186/s40064-016-1856-7 Raw data from the questionnaire reponses.

## Abbreviations

GUI: graphical user interface
HPC: high performance computing
ICT: information communication technology
UK: United Kingdom
