# Supplementary material for: Bringing computational science to the public
Source: Springerplus. 2016 Mar 2;5:259. doi: 10.1186/s40064-016-1856-7 (PMC4775721; doi:10.1186/s40064-016-1856-7)
Supplement: Supplementary file 1 — 10.1186/s40064-016-1856-7 Raspberry pi and bioinformatics in the Dundee science centre questionnaire. [file 40064_2016_1856_MOESM1_ESM.pdf]

<u>Raspberry pi and bioinformatics in the Dundee science centre questionnaire</u>

We would greatly appreciate your time to fill out the following questionnaire. Your responses to these questions are anonymous and you are free to decline to answer any question. This data will be used for evaluation of the workshop. The data may form some part of the ongoing research efforts to bring these workshops to larger audiences via scientific presentations (verbal and/or written) and future funding applications. This questionnaire is not for the Dundee science centre as a whole.

<u>General Questions</u> – *Please colour in the circle – LIKE this ● DO NOT tick or cross like this ⦻ ⊖ ✓*

Have you attended the Dundee science centre or another science centre previously? Yes O No O

Do you define yourself as Female, Male or Other? Female O Male O Other O

If other please specify if you wish - ________________________

How old are you? _____________

<u>Workshop related questions</u>

Please rank how far you agree with the following statements using the boxes provided.
Please colour in the circle – LIKE this ● DO NOT tick or cross like this ⦻ ⊖ ✓

Question	Strongly agree	Agree	Neutral	Disagree	Strongly disagree
I am interested in Science	O	O	O	O	O
Science experiments can be carried out on computers	O	O	O	O	O
I use a computer more than a tablet or other hand held device	O	O	O	O	O
Having attended the workshop today I am less excited by science	O	O	O	O	O
Having attended the workshop today I think computers are useful within biology	O	O	O	O	O
Having attended the workshop today I am more interested in programming a computer	O	O	O	O	O
Having attended the workshop today I am likely to use a Raspberry Pi in my own time	O	O	O	O	O
Having attended the workshop today I believe this type of workshop would be useful for students aged between 15 and 18	O	O	O	O	O

Please provide your own thoughts in the boxes provided

How easy was it to follow and understand the workshop today?

Do you have any suggestions to improve the workshop?

Thank you
